# Robust Sensing of Approaching Vehicles Relying on Acoustic Cues

**DOI:** 10.3390/s140609546

**Published:** 2014-05-30

**Authors:** Mitsunori Mizumachi, Atsunobu Kaminuma, Nobutaka Ono, Shigeru Ando

**Affiliations:** 1 Kyushu Institute of Technology, 1-1 Sensui-cho, Tobata-ku, Kitakyushu, Fukuoka 804-8550, Japan; 2 Nissan Motor Co., Ltd., 1-1 Aoyama, Morinosato, Atsugi, Kanagawa 243-0123, Japan; E-Mail: atunob-k@mail.nissan.co.jp; 3 National Institute of Informatics, 2-1-2 Hitotsubashi, Chiyoda-ku, Tokyo 101-8430, Japan; E-Mail: onono@nii.ac.jp; 4 The University of Tokyo, 7-3-1 Hongo, Bunkyo-ku, Tokyo 113-8656, Japan; E-Mail: ando@alab.t.u-tokyo.ac.jp

**Keywords:** active safety system, driver support system, acoustical sensing, spatio-temporal gradient method, particle filter

## Abstract

The latest developments in automobile design have allowed them to be equipped with various sensing devices. Multiple sensors such as cameras and radar systems can be simultaneously used for active safety systems in order to overcome blind spots of individual sensors. This paper proposes a novel sensing technique for catching up and tracking an approaching vehicle relying on an acoustic cue. First, it is necessary to extract a robust spatial feature from noisy acoustical observations. In this paper, the spatio-temporal gradient method is employed for the feature extraction. Then, the spatial feature is filtered out through sequential state estimation. A particle filter is employed to cope with a highly non-linear problem. Feasibility of the proposed method has been confirmed with real acoustical observations, which are obtained by microphones outside a cruising vehicle.

## 1. Introduction

Smart sensing technologies are widely used in modern vehicles. The latest developments in automobile design have allowed them to be equipped with a camera and a radar system, which are aimed at sensing people, obstacles, and other vehicles. Such sensors provide supplementary information to the driver. It is helpful for a driver to receive instructive information from these smart sensors. The sensing systems contribute not only to achieve active safety, but also to achieve driverless self-driving [1] and autonomous parking [2]. The equipped camera and radar systems may fail to capture the circumstances in some cases, where some barriers are on the traffic lane. For example, when a car comes to a blind junction of a highway, neither the camera nor the radar can detect the approaching cars in the main lane. On the other hand, acoustical noises, which are generated by the approaching cars, arrive at the car in the blind junction. In this paper, acoustical sensing of the approaching vehicle is proposed as an active safety system.

The acoustical signal is a suitable cue for recognizing an approaching car in blind conditions. However, the acoustical signal is sensitive to the presence of acoustical interferences. Another serious problem lies in the acoustical sensing of the approaching cars. To achieve the acoustical sensing, the vehicle must be equipped with external microphones to capture the acoustical signals. Therefore, the captured signals consist of the target signal, which is generated by the approaching car, and interferences such as wind noises and road traffic noises. It is necessary to robustly extract the target signal and localize the approaching car.

A robust spatial feature is required for achieving sound source localization with noisy observations. In this paper, the spatial feature is extracted by the spatio-temporal gradient method [3–5]. The spatio-temporal gradient method has an advantage of high temporal resolution with a non-iterative closed-form solution. It is difficult even for the spatio-temporal gradient method to accurately localize the approaching car with highly distorted observations. Filtering processes are indispensable for achieving robust sound localization. The Kalman filter can be also applied in a simple traffic condition, which can be described by a linear model [6]. In this paper, however, a non-linear particle filter [7] is employed as post-filtering. The particle filter has been widely applied in sound source localization under noisy environments [8–11], reverberant environments [12–14], noisy and reverberant environments [15–17], and multiple source conditions [18–22]. Those methods employ the conventional spatial features. The proposed method employs the advanced spatial feature, which is extracted by the spatio-temporal gradient method. The spatial feature is regarded as likelihood, and a random walk process is employed as a system model.

Feasibility of the proposed method is examined using real world data, when a target vehicle approaches the reference vehicle. The objective of the experiment is to catch and track the approaching vehicle, which comes from the rear side.

This paper is organized as follows: Section 2 overviews sound source localization, and Section 3 describes the robust spatial feature based on the spatio-temporal gradient method. Section 4 describes a state space model and sequential state estimation by particle filtering. In Section 5, the experimental setup is explained, and experimental results are shown to evaluate the feasibility of the proposed method. Finally, conclusions are given in Section 6.

## 2. Direction-of-Arrival Estimation

### 2.1. Overview

Spatial information on a sound source includes both direction of the source and distance to the source. Direction-of-arrival (DOA) estimation focuses only on estimating the direction. Sound source localization is the task of estimating both variables, namely, distance and direction to the source. In general, source localization requires a larger number of microphones when compared to DOA estimation.

**Figure 1.**
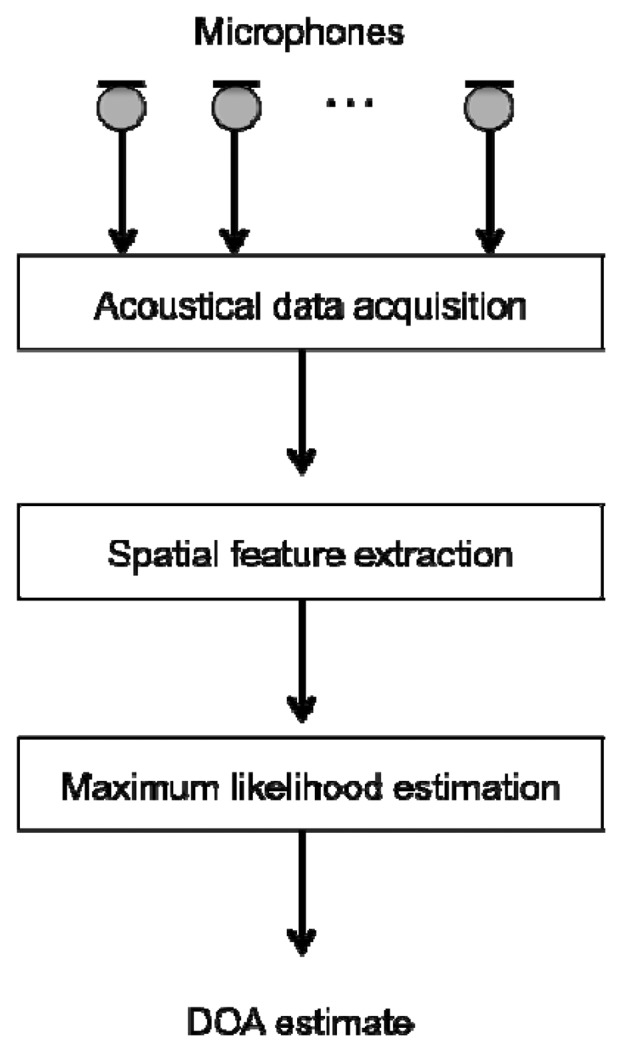
Outline of DOA estimation.

Figure 1 illustrates the architecture of a standard DOA estimator. A set of spatially-distributed microphones, that is, a microphone array, is usually used for obtaining the spatial information. A spatial feature for DOA estimation is extracted from the multi-channel observations captured by the spatially-distributed microphones. It is important that a robust spatial feature is provided for DOA estimation under adverse environments. DOA estimation is completed by peak search in the spatial feature.

DOA estimation can be achieved by various approaches. It is broadly divided into non-parametric and parametric methods. The parametric method uses a deterministic model, which describes the spatial relationship between a sound source and a microphone. Model parameters are determined based on a statistical fitting technique using less-distorted acoustical observations. Popular parametric DOA estimators are based on high-resolution spectral analysis such as a minimum variance algorithm [22], and a multiple signal classification (MUSIC) algorithm [23]. Those methods can yield the accurate DOA estimate, when the acoustical environment satisfies their assumptions. Those, however, fail in DOA estimation under non-stationary, heavy noisy, and high reverberant conditions.

### 2.2. Non-Parametric DOA Estimation

Concerning the non-parametric DOA estimation, beam scanning and time difference of arrival (TDOA) estimation are the two major techniques. The beam scanning technique relies on the difference in amplitude among multiple observations. The beam is formed by delay-and-sum beamforming [24], and the main-lobe is steered in the search space. The most dominant steered direction, *i.e.,* the one that returns the highest energy in the beamformer output, is regarded as the DOA estimate. The beam scanning can be performed with small computational complexity, but is not robust against background noise and room reverberation. It also requires a large-scale microphone array to form a sharp main-lobe in delay-and-sum beamforming [25].

TDOA estimation is widely employed in DOA estimation using a small-scale microphone array such as a paired-microphone. In 2-ch TDOA estimation, stereo observations acquired by a paired-microphone are defined as follows:

(1) $$x_{1}(t)=h_{1}(t)*s(t)+n_{1}(t)$$

(2) $$x_{2}(t)=h_{2}(t)*s(t)+n_{2}(t)$$

where *s*(*t*) is a target source signal, *h_i_*(*t*) is the room impulse response between the target source and *i*-th microphone, *n_i_*(*t*) is a channel-dependent background noise, and * means a convolution operation, respectively. The acoustical condition in the room is assumed as linear time-invariant. In the free field, that is, a non-reverberant sound field, Equations (1) and (2) are simply written as follows:

(3) $$x_{1}(t)=a_{1}s(t-\tau_{1})+n_{1}(t)$$

(4) $$x_{2}(t)=a_{2}s(t-\tau_{2})+n_{2}(t)$$

where *a_i_* is a constant attenuation factor, and *τ_i_* is the propagation time, when the target signal arrives at each microphone. TDOA *τ*_12_ can be estimated based on the phase difference between two observations, *x*_1_(*t*) and *x*_2_(*t*):

(5) $$\tau_{12}\equiv \tau_{1}-\tau_{2}$$

Cross correlation *r*_12_(*τ*) between the stereo observations is the most popular spatial feature for the TDOA estimation:

(6) $$r_{12}(\tau )=\frac{1}{T}\int_{0}^{T}x_{1}(t)x_{2}(t+\tau )dt$$

The TDOA estimate is given as with the maximum of the cross correlation *r*_12_(*τ*):

(7) $${\hat{\tau }}_{12}=\underset{\tau }{\text{argmax}}\left[r_{12}\left(\tau \right)\right]$$

In general, phase difference is much robust against acoustical interferences than amplitude difference. Therefore, the cross correlation is modified in TDOA estimation. The generalized cross correlation [26] is widely used in TDOA estimation:

(8) $${\tilde{r}}_{12}(\tau )=\int_{-\infty }^{\infty }\frac{X_{1}\left(\omega \right){X_{2}}^{*}\left(\omega \right)}{\left|X_{1}\left(\omega \right)\right|\left|X_{2}\left(\omega \right)\right|}e^{j\omega \tau }d\omega$$

where *X*_1_(ω) and *X*_2_(ω) are the Fourier transform of the stereo observation, *x*_1_(*t*) and *x*_2_(*t*), and * represents the complex conjugate:

(9) $$X_{1}\left(\omega \right)=\int_{-\infty }^{\infty }x_{1}\left(t\right)e^{-j\omega t}dt$$

(10) $$X_{2}\left(\omega \right)=\int_{-\infty }^{\infty }x_{2}\left(t\right)e^{-j\omega t}dt$$

In Equation (8), the spatial feature is based on the phase transform, and is robust against noise and reverberation [27,28]. The smoothed coherence transformation [29] is also a well-known robust spatial feature. In case of single dimensional space, the DOA estimate is straightforwardly given by the TDOA estimate as follows:

(11) $$\hat{\theta }={\text{sin}}^{-1}\frac{{\hat{\tau }}_{12}c}{d}$$

where *c* is the sound velocity, and *d* is the microphone spacing.

## 3. Robust Spatial Feature

It is important for DOA estimation to use a suitable spatial feature, which is robust against acoustical interferences such as noise and reverberation. Acoustical observations obtained by vehicle-mounted microphones outside the vehicle are heavily distorted, and then the traditional spatial features are not appropriate for this purpose. In this section, a robust spatial feature is introduced for DOA estimation with heavily distorted observations.

The spatio-temporal gradient method has been proposed for 3-D sound source localization based on the spatio-temporal derivative of multi-channel acoustic signals. The principle of the spatio-temporal gradient has been originally applied into image processing, but is compatible with sound source localization on the spatio-temporal domain [3–5].

Let us assume that sound pressure of a point source is observed as *f*(*t*) at a microphone position. Spatial and temporal gradients of the sound pressure *f*(*t*) are written in 3D sound space as *f_x_*(*t*), *f_y_*(*t*), *f_z_*(*t*), and *f_t_*(*t*), respectively. The relationship among sound pressure, its spatial and temporal gradients, is given as follows [5]:

(12) $$f_{x}\left(t\right)-\frac{u_{x}}{R}f\left(t\right)-\frac{u_{x}}{c}f_{t}\left(t\right)=0$$

(13) $$f_{y}\left(t\right)-\frac{u_{y}}{R}f\left(t\right)-\frac{u_{y}}{c}f_{t}\left(t\right)=0$$

(14) $$f_{z}\left(t\right)-\frac{u_{z}}{R}f\left(t\right)-\frac{u_{z}}{c}f_{t}\left(t\right)=0$$

where **u** = (*u_x_*, *u_y_*, *u_z_*) is the unit vector from the observation point to the sound source, *R* is the distance between the observation point and the sound source, and *c* is the sound velocity, respectively. Therefore, **u** and *R* mean the direction of the target source and the source distance, respectively. In this paper, a single dimensional DOA estimation is carried out in [0 deg., 180 deg.] with stereo observations. The scalar component *u_x_* is estimated in Equation (12), and then a DOA estimate *θ* is formally given by *θ* = sin^−1^*u_x_*. In the process of DOA estimation, the sequence of an observation is segmented into framed data using a window function, *w*(*t*), of which length is *T*. In Equation (12), a weight function is multiplied into the framed data, and the weighted equation is integrated in [*0*, *T*]:

(15) $$F_{x}\left(\tau ,\omega \right)-\frac{u_{x}}{R}F\left(\tau ,\omega \right)-\frac{u_{x}}{c}F_{t}\left(\tau ,\omega \right)=0$$

where:

(16) $$F\left(\tau ,\omega \right)=\int_{0}^{T}f\left(t+\tau \right)w(t)e^{-j\omega t}dt$$

(17) $$F_{x}\left(\tau ,\omega \right)=\int_{0}^{T}f_{x}\left(t+\tau \right)w(t)e^{-j\omega t}dt$$

(18) $$F_{t}\left(\tau ,\omega \right)=\int_{0}^{T}f\left(t+\tau \right)\left\{j\omega w(t)-w_{t}(t)\right\}e^{-j\omega t}dt$$

Here, *F_t_*(*τ*,*ω*) depends on *w*(*t*) and its temporal gradient *w_t_*(*t*). Spatio-temporal information is represented in Equation (15) regardless of the window length *T*. Both *u_x_*(*τ*) and *R*(*τ*) are given in [0 deg., 180 deg.] as the least square solutions in the temporal-spectral domain as follows [4]:

(19) $$u_{x}\left(\tau \right)=\frac{\underset{\omega }{\text{∑}}{\left|F\left(\tau ,\omega \right)\right|}^{2}\underset{\omega }{\text{∑}}\text{Re}\left[F_{x}{\left(\tau ,\omega \right)}^{*}F_{t}\left(\tau ,\omega \right)\right]-\underset{\omega }{\text{∑}}\text{Re}\left[F{\left(\tau ,\omega \right)}^{*}F_{x}\left(\tau ,\omega \right)\right]\underset{\omega }{\text{∑}}\text{Re}\left[F{\left(\tau ,\omega \right)}^{*}F_{t}\left(\tau ,\omega \right)\right]}{\underset{\omega }{\text{∑}}{\left|F\left(\tau ,\omega \right)\right|}^{2}\underset{\omega }{\text{∑}}{\left|F_{t}\left(\tau ,\omega \right)\right|}^{2}-{\left(\underset{\omega }{\text{∑}}\text{Re}\left[F{\left(\tau ,\omega \right)}^{*}F_{t}\left(\tau ,\omega \right)\right]\right)}^{2}}$$

(20) $$R\left(\tau \right)=\frac{\underset{\omega }{\text{∑}}{\left|F\left(\tau ,\omega \right)\right|}^{2}\underset{\omega }{\text{∑}}\text{Re}\left[F_{x}{\left(\tau ,\omega \right)}^{*}F_{t}\left(\tau ,\omega \right)\right]-\underset{\omega }{\text{∑}}\text{Re}\left[F{\left(\tau ,\omega \right)}^{*}F_{x}\left(\tau ,\omega \right)\right]\underset{\omega }{\text{∑}}\text{Re}\left[F{\left(\tau ,\omega \right)}^{*}F_{t}\left(\tau ,\omega \right)\right]}{\underset{\omega }{\text{∑}}\text{Re}\left[F{\left(\tau ,\omega \right)}^{*}F_{x}\left(\tau ,\omega \right)\right]\underset{\omega }{\text{∑}}{\left|F_{t}\left(\tau ,\omega \right)\right|}^{2}-\underset{\omega }{\text{∑}}\text{Re}\left[F{\left(\tau ,\omega \right)}^{*}F_{t}\left(\tau ,\omega \right)\right]\underset{\omega }{\text{∑}}\text{Re}\left[F_{x}{\left(\tau ,\omega \right)}^{*}F_{t}\left(\tau ,\omega \right)\right]}$$

Both *u_x_*(*τ*) and *R*(*τ*) are sequentially updated using the time-variant observations in short-term frames.

In this paper, only the DOA estimate *u_x_*(*τ*) is used for sensing the approaching vehicle. The spatial gradient, *f_x_*(*t*), is defined as the difference between stereo observations. A pair of free-field response microphones is used for calculating the sound pressure and its spatial gradient. In practical, Equation (15) is solved in the frequency domain. The DOA estimate is given in each frequency. Low frequency components are distorted by acoustical interferences, and then are ignored in DOA estimation. The selected DOA estimates forms the DOA histogram in each short-term frame.

## 4. Particle Filtering

### 4.1. State Space Model

The spatial feature is provided by the spatio-temporal gradient method with stereo observations, **x**(*t*) = (*x*_1_(*t*), *x*_2_(*t*)), which are noisy signals observed by two spatially-separated, vehicle-mounted microphones. The spatial feature can be regarded as a probability distribution for DOA existence on single-dimensional state space in [0 deg., 180 deg.]. DOA estimate is given as the direction with the maximum in the spatial feature *p*(*θ*|**x**):

(21) $$\hat{\theta }=\underset{\theta }{\text{argmax}}\left\{p\left(\theta |x\right)\right\}$$

Difficulty in DOA estimation is caused by distortion on the spatial feature *p*(*θ*|**x**) due to various kinds of noises.

### 4.2. DOA Estimation through State Estimation

In the scenario of the traffic scene around the junction of the highway, it is difficult to model a DOA, which is determined by a relationship between the motion of a reference vehicle and independent movements of surrounding vehicles. Roughly speaking, however, the DOA must change smoothly in between short-term frames. As a system model, a random walk process is applied to model the stochastic behavior of the DOA as follows:

(22) $$\theta_{k}=\theta_{k-1}+\nu_{k},\nu_{k}\sim N\left(0,\sigma^{2}\right)$$

where *θ_k_* represents the true DOA at the *k* -th time frame, and *ν* means the zero-mean Gaussian noise with the variance σ^2^. The true DOA trajectory and the sampled observations up to the *k* -th frame are noted as follows:

(23) $$\begin{array}{l} \theta_{1:k}=\left\{\theta_{1},\theta_{2},\text{L},\theta_{k}\right\}\\ x_{1:k}=\left\{x_{1},x_{2},\text{L},x_{k}\right\} \end{array}$$

The spatial feature can be regarded as likelihood *p*(**x***_k_*|*θ_k_*). State estimation is formally done in a recursive form of the posterior distribution, *p*(*θ*_1:_*_k_*|**x**_1:_*_k_*), as follows:

(24) $$p\left(\theta_{1:k}|x_{1:k}\right)\propto p\left(\theta_{1:k-1}|x_{1:k-1}\right)p\left(x_{k}|\theta_{k}\right)p\left(\theta_{k}|\theta_{k-1}\right)$$

### 4.3. Particle Filtering

Sequential state estimation is done by particle filtering in the Bayesian framework [7]. We employ a bootstrap filter, which uses the system model as proposal distribution [7]. DOA estimation is performed with the posterior spatial feature by particle filtering. In practice, weighted particles are sequentially updated according to Equation (24). In the initial frame, particles {*θ*_0_^(*l*)^} (*l* = 1,2,⋯, *M*) with the same weight 1/*M* are drawn from uniform distribution in [0 deg., 180 deg.]. Particles at the *k*-th frame are drawn from the system model in Equation (22), and the weight for each particle is updated by the likelihood as follows:

(25) $$\begin{array}{r} \theta_{k}^{\left(l\right)}\sim p\left(\theta_{k}|\theta_{k-1}^{\left(l\right)}\right)\\ w_{k}^{\left(l\right)}=p\left(x_{k}|\theta_{k}^{\left(l\right)}\right) \end{array}$$

where *l* = 1,2,⋯, *M*. The particles {*θ_k_*^(*l*)^} (*l* = 1,2,⋯, *M*) are sampled with replacement in proportion to the weight {*θ_k_*^(*l*)^} (*l* = 1,2,⋯, *M*) . The resampled particles are used as the proposal particle distribution in the next frame. DOA is estimated by finding the peak of the filtered spatial feature. The peak is obtained by averaging the weighted particles. In the case with a small set of particles, the spatial feature is obtained from the weighted particles convolved with Gaussian kernels.

## 5. Performance Evaluation

### 5.1. Experimental Scenario

**Figure 2.**
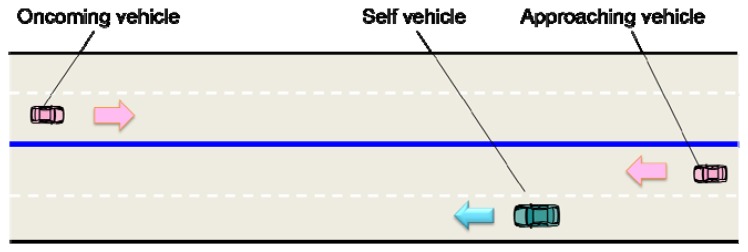
Traffic scene with the reference vehicle with microphones and two approaching vehicles in the same and the opposite lane, respectively. The reference vehicle (middle-size sedan) ran in a cruising lane, and the target vehicle (hatchback) approached from the rear in the passing lane. Another oncoming vehicle (large-size sedan) passed along the opposite lane.

The relative DOA between a reference vehicle and an approaching vehicle coming from the rear side was estimated, when an oncoming vehicle also existed in the opposite lane. Figure 2 shows the outline of the experimental field. The reference middle-size sedan (self vehicle) cruises equipped with several microphones, when a hatchback approaches the reference vehicle from the rear and a large-size sedan approaches in the oncoming lane. The reference vehicle is constantly moving at the speed of 30 km/h, and the approaching vehicle from the rear is moving at 50 km/h. In other words, the relative speed between the reference vehicle and the approaching vehicle from the rear is set at 20 km/h. The oncoming vehicle approaches at the speed of 50 km/h in the opposite lane. Data collection was carried out several times in the same traffic scenario.

### 5.2. Data Preparation

**Figure 3.**
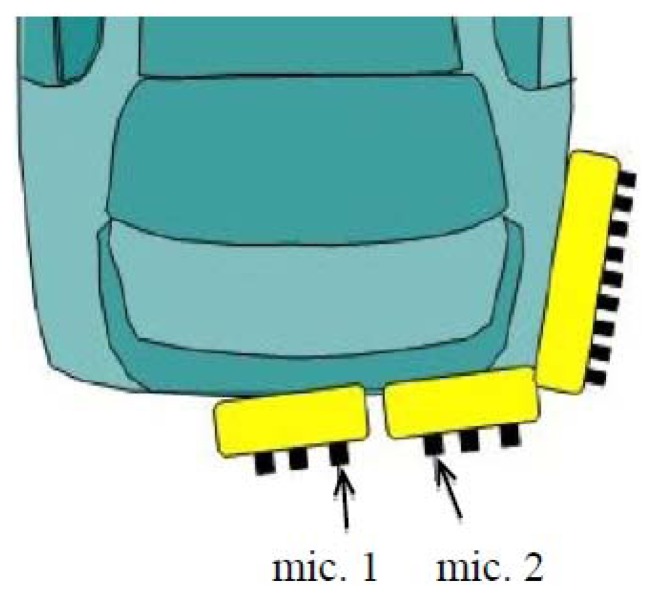
Microphone arrangement for sensing approaching vehicles from the rear. Two microphones (mic. 1 and mic. 2) were empirically selected out for DOA estimation.

In this experiment, the target was the approaching vehicle from the rear. Thus, microphones were installed at the back of the vehicle. Microphone arrangement was also considered to efficiently capture the approaching vehicle. In this experiment, 15 calibrated microphones (SONY ECM-77B) were put on the rear side as shown in Figure 3. In practical, a pair of microphones was empirically selected out for DOA estimation. The spacing between the microphones was 74 mm.

The observed signals were sampled at 48 kHz with 16 bits accuracy. The DOA histogram was calculated in each frame, of which length was set at 1024 samples. In each frequency bin, of which width was 46.8 Hz, a DOA estimate was given by the spatio-temporal gradient method. The DOA estimates in the frequency range from 200 Hz to 15,000 Hz formed the DOA histogram. The width of the DOA histogram bin was set at 10 degrees. A narrower width gives a DOA estimate in high resolution, but requires a higher computational cost. For an active safety system, the realization of the real-time processing has precedence over the accuracy of the DOA estimate.

**Figure 4.**
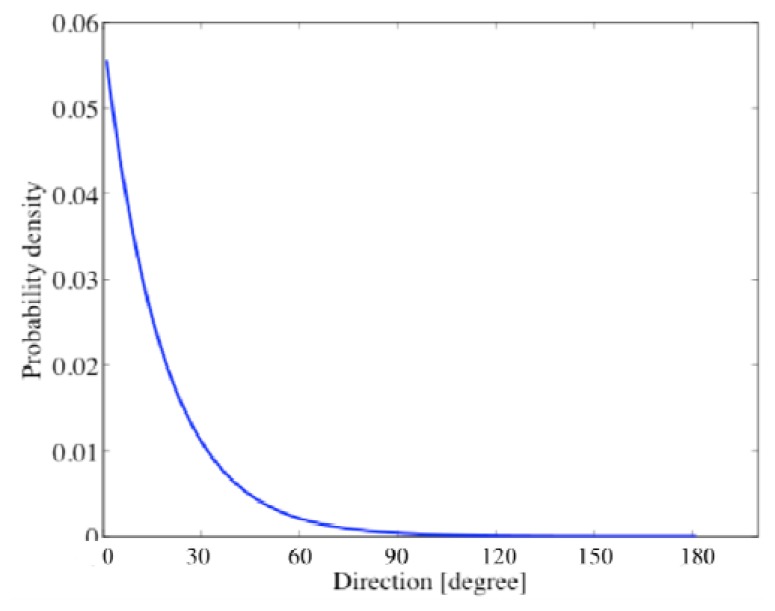
Exponential probability distribution used for determining particle arrangement in the initial frame.

The particle filter employed 100 particles in the DOA range of [0 deg., 180 deg.]. The variance *σ*^2^ of the system noise in Equation (22) was empirically set at one degree. Likelihood in particle filtering was given by averaging those weighted particles. Resampling was carried out in each frame in order to avoid degradation of particles. It is important for particle filtering to appropriately arrange the particles in the initial frame [30]. In general, the initial particles shall be uniformly distributed in [0 deg., 180 deg.] without a priori information. In the scenario in Figure 2 where the approaching target vehicle is located at 0 degree approximately, the initial particles should be distributed in proportion to the exponential distribution as shown in Figure 4. The parameter of the exponential function is determined assuming vehicles in forward direction are not considered in the acoustical sensing.

### 5.3. Experimental Results

DOA estimation was carried out using the stereo observations. When the target vehicle was far from the reference vehicle, the observation did not include sufficient information on the target vehicle. When the energy of the acoustical observation exceeded a threshold, DOA estimation began automatically. The threshold was empirically determined in this experimental scenario. True DOA trajectories were obtained using a GPS system, of which sampling frequency was set at 20 kHz. Three sets of different scene (Scenes 1–3) were used for DOA estimation.

**Figure 5.**
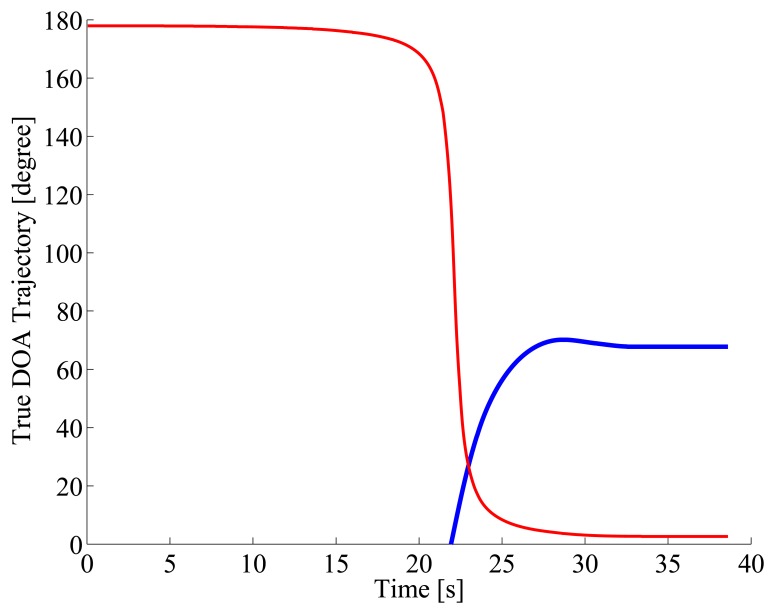
True DOA trajectories are drawn for the target vehicle approaching from the rear (blue line) and the oncoming vehicle in the opposite lane (red line), respectively. The target vehicle approaches from the rear (0 degree) and runs abreast with the reference vehicle in the same lane, after the oncoming vehicle passes along the opposite lane.

**Figure 6.**
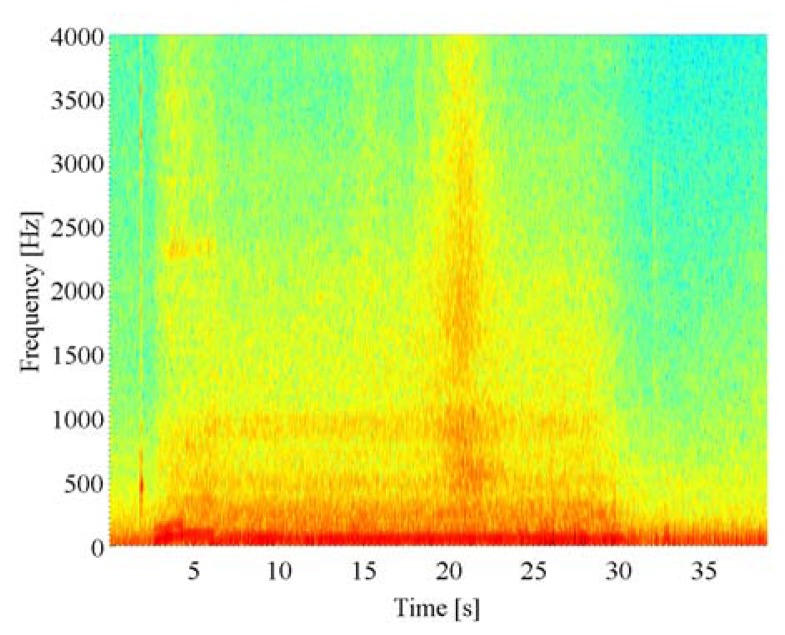
Spectrogram obtained from the acoustical observation by the reference vehicle.

**Figure 7.**
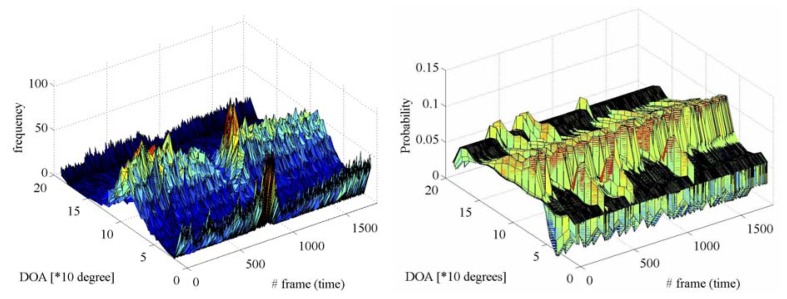
Spatial features, which are obtained by the spatio-temporal gradient method and the conventional cross-correlation-based method, are displayed in left and right panels, respectively.

Figure 5 gives the true DOA trajectories in Scene 1. 0 degree, 90 degrees, and 180 degrees indicate backward, side, and forward directions of the reference vehicle, respectively. The DOA trajectory of the approaching vehicle from the rear as the target for acoustical sensing is drawn with a blue line, and that of the oncoming vehicle in the opposite lane as the interference is drawn with a red line. Figure 6 shows the spectrogram of the acoustical observation, which is obtained by using the microphone mounted on the reference vehicle. Figure 7 displays the spatial features, which are obtained by the spatio-temporal gradient method in Equation (19) and the conventional cross-correlation-based method in Equation (8), in left and right panels, respectively. It is impossible to achieve DOA estimation with the conventional cross-correlation-based method. Therefore, the cross-correlation-based spatial feature could not be adopted as the likelihood in particle filtering.

**Figure 8.**
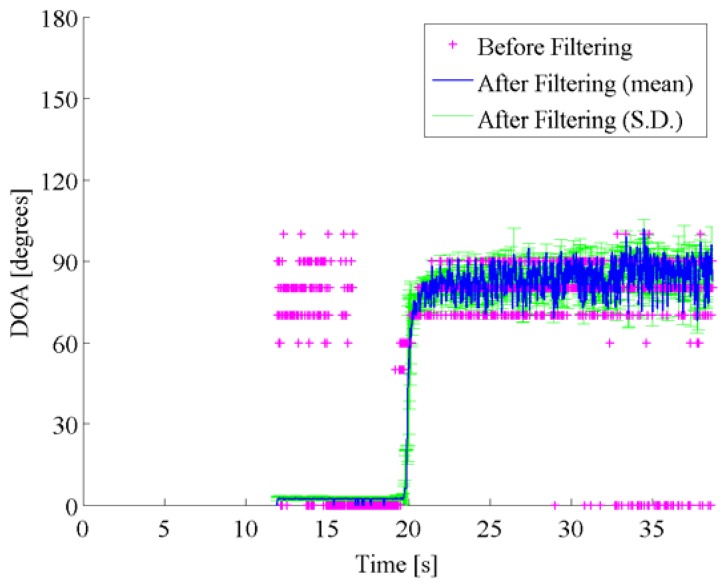
DOA estimates are shown for pre-filtered DOA candidates (pink cross marks) with the DOA histogram shown in the left panel in Figure 7, mean (blue solid line) and standard deviation (green error bars) among post-filtered DOA estimates over 1000 runs on Scene 1, respectively.

Figure 8 shows both the pre-filtered DOA estimates by the spatio-temporal gradient method and the post-filtered DOA estimates by particle filtering. In Figure 8, the pre-filtered DOA estimates are obtained as the DOA with the maximum of the DOA histogram in each frame, and are represented by the pink cross marks. The post-filtered DOA trajectory, which is represented by the blue line, is averaged over 1000 runs in particle filtering, where the same likelihood is used with the same initial particle distribution. Figure 8 also displays the standard deviation among the post-filtered DOA estimates over 1000 runs by error bars.

**Figure 9.**
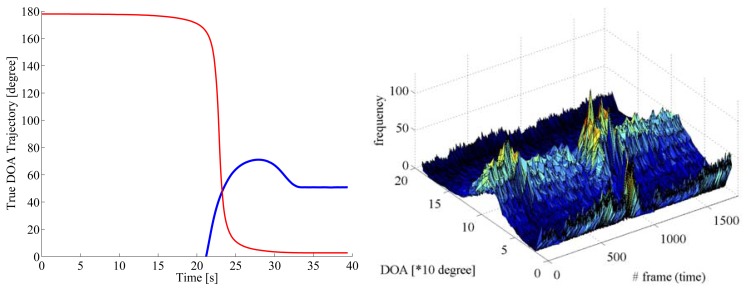
Left panel shows true DOA trajectories in Scene 2 for the target vehicle approaching from the rear (blue line) and the oncoming vehicle in the opposite lane (red line), respectively. Right panel displays pre-filtered DOA histogram. The target vehicle approaches from the rear (0 degree) and separates from the reference vehicle, while the oncoming vehicle passes along the opposite lane.

**Figure 10.**
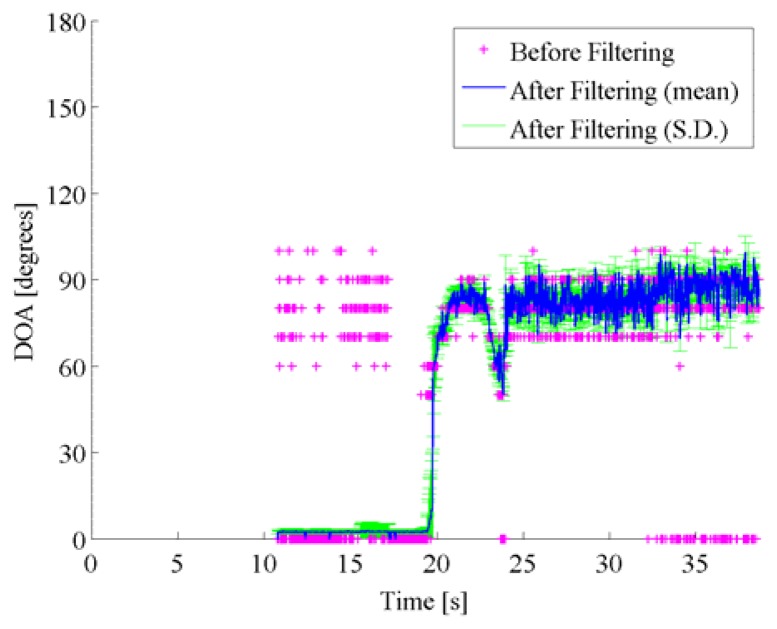
DOA estimates are shown for pre-filtered DOA candidates (pink cross marks) with the DOA histogram shown in the right panel in Figure 9, mean (blue solid line) and standard deviation (green error bars) among post-filtered DOA estimates over 1000 runs on Scene 2, respectively.

**Figure 11.**
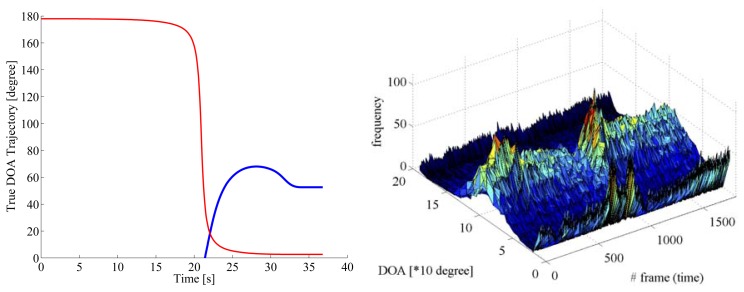
Left panel shows true DOA trajectories in Scene 3 for the target vehicle approaching from the rear (blue line) and the oncoming vehicle in the opposite lane (red line), respectively. Right panel displays pre-filtered DOA histogram. The target vehicle approaches from the rear (0 degree) and separates from the reference vehicle, while the oncoming vehicle passes along the opposite lane.

**Figure 12.**
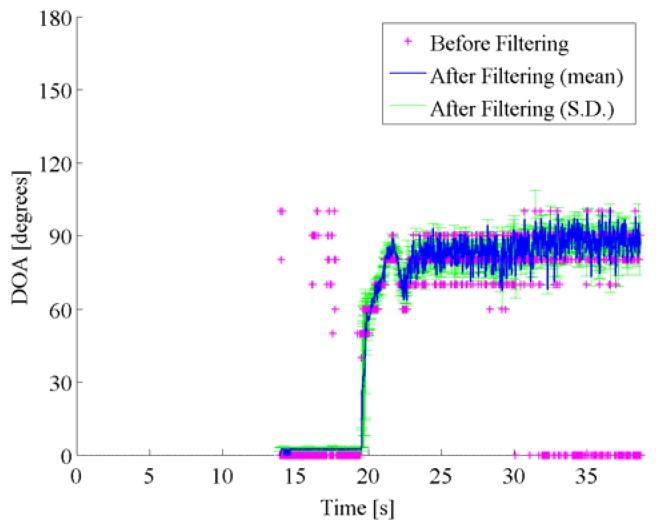
DOA estimates are shown for pre-filtered DOA candidates (pink cross marks) with the DOA histogram shown in the right panel in Figure 11, mean (blue solid line) and standard deviation (green error bars) among post-filtered DOA estimates over 1000 runs on Scene 3, respectively.

Concerning the data shown in Figure 8, the pre-filtered DOA histograms have peaks around 90 degrees in the beginning up to 17 s approximately, although no vehicle existed at the side. In this scenario, the approaching vehicle chased the reference vehicle, and ran abreast with each other. It is supposed that those peaks in the DOA histograms correspond to the directions of the noise sources such as the engine noise, the exhaust noise, the tire noise, and the wind noises related to the reference vehicle. Alternative peaks around 0 degree corresponds to the noises caused by the approaching vehicle from the rear, that is, 0 degree. Those peaks around 0 degree dominated, as the target vehicle approached. The particle filter contributed to accurately disregard the DOA candidates caused by acoustical interferences. In Scenes 2 and 3, the true DOA trajectories and the spatial features obtained by the spatio-temporal gradient method are given in Figures 9 and 11, and pre-filtered and post-filtered DOA estimates are shown in Figures 10 and 12, respectively.

In Scene 2 as shown in Figures 9 and 10, the target vehicle approached and separated, while the oncoming vehicle passes along the opposite lane. Therefore, the filtered results were influenced by the acoustical noises caused by the ongoing vehicle in the opposite lane. As the results, the estimated DOA trajectories have sharp dips around 24 s in Figure 10. The post-filtered results are stuck to 80 degrees, although the target vehicle separates from the reference vehicle after 30 s. It is considered that noises from the reference vehicle have generated a ghost sound source at the direction around 80 degrees.

To improve the tracking performance, particle transition according to asymmetrical probability distribution should be substitute for the random walk model with the Gaussian system noise. In Scene 3 as shown in Figures 11 and 12, it tends to be similar to the results in Scenes 1 and 2. Those DOA estimation results are summarized in Table 1. Table 1 gives the means and the standard deviations among the errors of the pre-filtered and post-filtered DOA estimates over frames.

The DOA estimation errors are relatively large, because the spatial resolution of the spatial feature is set to 10 degrees. The average error over the post-filtered DOA estimates is 10 degrees smaller than that of the pre-filtered DOA candidates. An advantage of particle filtering depends on a traffic scene. At least, the filtering could reduce the error in DOA estimation in 5 degrees. In total, the proposed method succeeds in capturing and tracking the approaching target vehicle from the rear. In particle filtering, a real-time factor was 0.096 using a 2.6 GHz Intel Core i7 processor. It means that the filtering process can be done in real time.

## 6. Conclusions

It is important to achieve robust sensing of surrounding vehicles in order to design an active safety system. In this paper, a novel sensing method relying on acoustic cues is proposed to detect and track a vehicle approaching from the rear side. The direction of the approaching vehicle was estimated through the sequential state estimation with the robust spatial feature, which was extracted by the spatial-temporal gradient method. Performance of the proposed method has been confirmed with real world data, which were obtained by the vehicle-mounted microphones outside the vehicle. The proposed method succeeded in estimating the direction of the approaching vehicle from the rear in real time. It was impossible for a conventional cross-correlation-based spatial feature to achieve DOA estimation, but the spatial-temporal gradient method delivered reasonable DOA candidates. The particle filter contributed in reducing the estimation errors by 10 degrees in average. Future works include performance evaluation under more complicated traffic scenes.

## Figures and Tables

**Table 1.** Mean errors and standard deviations of the pre-filtered and post-filtered DOA estimates.

Scene	Pre-Filtered DOA Candidates	Post-Filtered DOA Estimates
	
	Mean (Standard Deviation) [degrees]	Mean (Standard Deviation) [degrees]
Scene 1	30.3 (22.8)	25.3 (23.4)
Scene 2	31.5 (23.0)	21.3 (19.8)
Scene 3	37.2 (26.4)	22.5 (20.9)
Averaged over all Scenes	33.0 (24.0)	23.0 (21.4)
